# The transcriptional regulator Sin3a activates CD44 and promotes collective luminal breast cancer cell migration

**DOI:** 10.1016/j.jbc.2025.110264

**Published:** 2025-05-21

**Authors:** Yaqi Qiu, Guangxin Luan, Yiwen Liu, Yiqing He, Guoliang Zhang, Qian Guo, Cuixia Yang, Yan Du, Feng Gao

**Affiliations:** 1 Department of Molecular Biology, Shanghai Sixth People's Hospital Affiliated to Shanghai Jiao Tong University School of Medicine, Shanghai, China; 2 Department of Clinical Laboratory, Shanghai Sixth People's Hospital Affiliated to Shanghai Jiao Tong University School of Medicine, Shanghai, China

**Keywords:** CD44, collective migration, lamellipodia, leader cell, Sin3a

## Abstract

Luminal type breast cancer (BrCa) cells invade into the surrounding tissues as collective strands, making them more metastatic than single cells. We have previously reported that the leading subpopulation of collective cells expressed high levels of CD44, which was associated with enhanced migratory and invasive potential of BrCa. It is crucial to elucidate how CD44 becomes enriched in leader cells and contributes to collective migration. In this study, we aimed to uncover the mechanisms responsible for CD44 upregulation in this context. First, we demonstrated that CD44 could facilitate dynamic lamellipodia formation by interacting with cytoskeletal proteins through its cytoplasmic domain. Then, we identified that a transcriptional regulator, Sin3a, was remarkably upregulated at the front edge of collectively migrating cells, exhibiting a correlation with enhanced CD44 expression. Notably, the knockdown of Sin3a effectively suppressed CD44 enrichment and lamellipodia outgrowth in leader cells, resulting in a significantly decreased cohesive movement of BrCa cells *in vitro* and *in vivo*. Our findings suggested that Sin3a was a novel regulator in CD44-facilitated lamellipodia formation and subsequent collective migration. This study elucidated the molecular mechanism underlying CD44 upregulation during collective migration of luminal-type BrCa cells, providing potential therapeutic targets to prevent cancer metastasis.

Collective cell migration is a crucial driver of coordinated multicellular movements widely observed in cancer metastasis, including breast cancer (BrCa) (1, 2). Notably, collectively migrating cancer cells exhibit higher metastatic potential than individual ones. During this process, the leading edge at the front of the collective clusters contributes to the driving force for the movement of the group of cells (1). However, the underlying mechanisms remain unclear.

Cohesive migration comprises functionally distinct cell populations, where leading cells localized at the front edge orchestrate the coordinated movement of the following cells (3, 4). The cellular disparity may be attributed to the differential activation of molecular pathways and transcriptional regulations between leader and follower cells. Previous reports demonstrated that leader cells exhibit elevated expression of keratin 14 and p63, which trigger collective invasion (5). Another study indicated that cells with high expression of myosin X can function as leader cells and drive the coordinated migration in lung cancer (6). Furthermore, we previously reported that CD44 is enriched in the leader cells of the luminal-type BrCa, whereas the followers maintain at a relatively low level of CD44 (7). As a cancer stem cell marker, CD44 has been found to play a crucial role in promoting cancer cell metastasis (8, 9, 10, 11). Given that leader cells frequently exhibit to be more motile and invasive than followers (12), it is reasonable to speculate that CD44 enrichment in leader cells may be indispensable for cancer cell collective migration.

Cancer cell movement relies on the increased actin-rich protrusion formation, including lamellipodia, filopodia, and invadopodia (13, 14). Significantly, lamellipodia, characterized as thin and sheet-like extensions at cell membrane, are regarded as the dominant protrusions in leader cells that drive the movement of follower cells during collective migration (15, 16). It has been reported that CD44 is highly concentrated in the lamellipodia of lung cancer cells (17). Similarly, our previous study demonstrated that CD44 is enriched in lamellipodia of luminal-type BrCa cells (18). These findings suggest that CD44 may be involved in lamellipodia formation during cancer metastasis. However, the mechanisms underlying CD44 enrichment at leader cells and its contribution to lamellipodia formation remain largely unknown. Indeed, it is well established that the upstream transcription factors could regulate CD44 expression in cancer cells. For instance, studies have demonstrated that CD44 expression is modulated by the tumor suppressor p53 and its paralog p63 in mammary epithelial cells with high tumorigenic potential (19). Moreover, KLF4-mediated transcriptional repression of CD44 has been shown to suppress stemness properties and metastatic potential of pancreatic cancer cell (20). These findings prompt us to speculate that specific transcriptional regulators of CD44 may be active at the leading edge of BrCa cells during collective migration.

In this study, we showed that the transcriptional factor Sin3a was upregulated in luminal-type BrCa leader cells, which could enhance CD44 expression. Importantly, we found that the knockdown of Sin3a could impair the lamellipodia formation and attenuate the collective migration. These findings illustrated a function of Sin3a in orchestrating CD44 accumulation in leader cells of luminal-type BrCa, suggesting a possible therapeutic target in the future.

## Results

### CD44-enriched leader cells enhance collective migration in luminal-type BrCa cells

**Figure 1:**
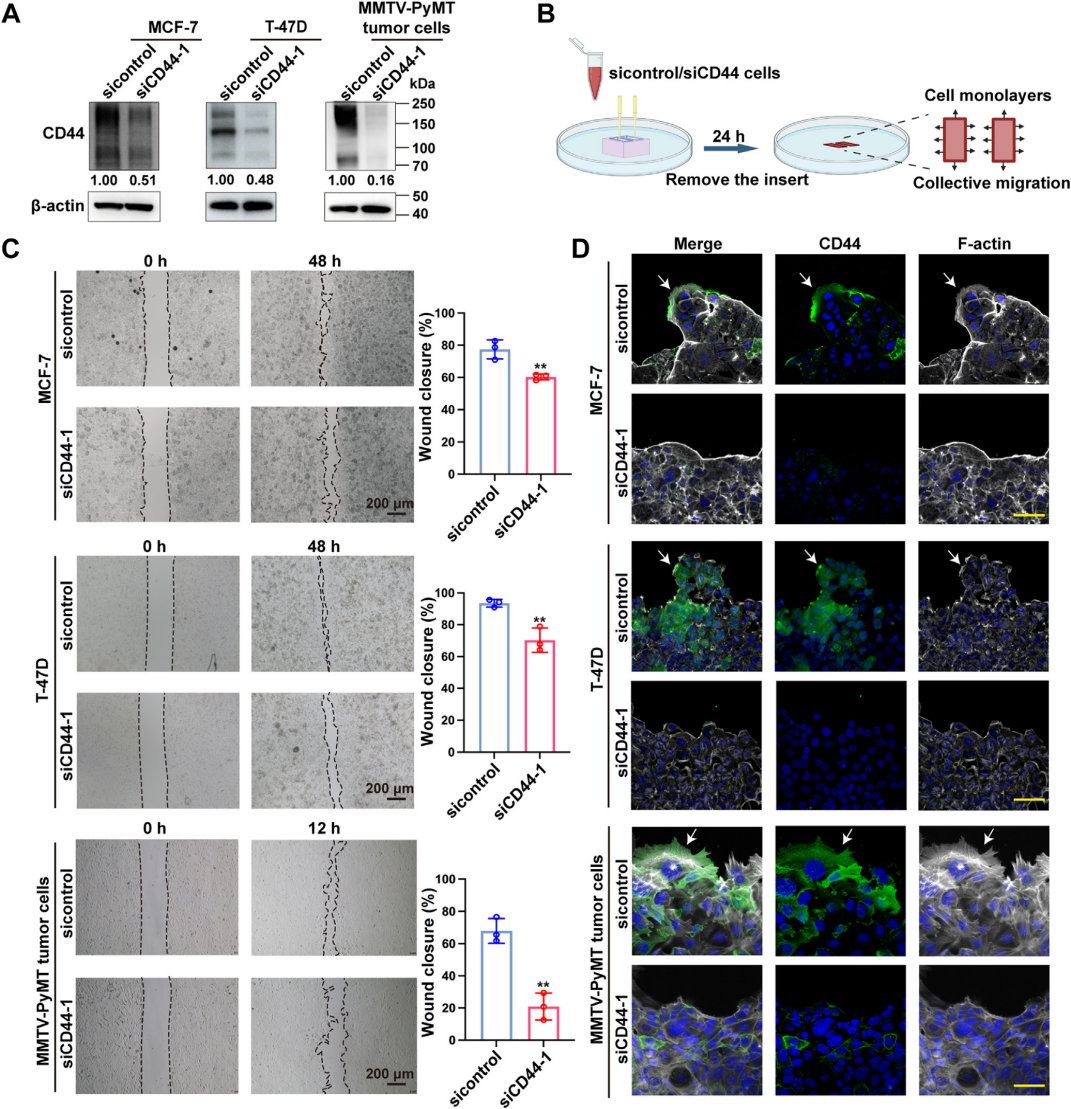
**CD44-enriched leader cells enhance collective migration in the luminal-type BrCa cells.***A*, Western blot analysis of CD44 levels in control *versus* CD44-knockdown MCF-7 cells, T-47D cells, and MMTV-PyMT tumor cells. *B*, schematic illustration of the *in vitro* wound healing assay (created with BioRender.com). The control and CD44-knockdown cells were independently cultured in adjacent wells separated by a defined cell-free gap. After confluency, the silicone insert was removed to trigger collective migration. *C*, representative images and quantitative analysis of wound healing assays in control *versus* CD44-knockdown MCF-7 and T-47D cells and MMTV-PyMT tumor cells. Wound closure rates (%) were presented as mean ± SD from three independent experiments. Scale bars represent 200 μm. ∗∗*p* < 0.01. *D*, immunofluorescence staining of CD44 (*green*) and F-actin (*white*) during collective migration in control *versus* CD44 knockdown MCF-7 and T-47D cells and MMTV-PyMT tumor cells. Scale bars represents 50 μm. BrCa, breast cancer.

We previously reported that CD44 was highly expressed in the leader cells of luminal-type BrCa; however, its role in collective migration remains to be clarified. To this end, we knocked down CD44 in both human BrCa cell lines (MCF-7 and T-47D) and primary tumor cells isolated from MMTV-PyMT spontaneous BrCa mouse model (Fig. 1*A*). The wound healing assays showed that the cooperative migration was markedly reduced in CD44-silenced cells (Fig. 1, *B* and *C*). As reported previously, leading cells at the forefront of collective migration are characterized by the outgrowth of lamellipodia (21), we next investigated whether CD44 localizes to lamellipodia in BrCa leader cells. As shown in Figure 1*D*, CD44 was enriched in the lamellipodia at the leading edge. Notably, the knockdown of CD44 dramatically suppressed the lamellipodia outgrowth. These findings were confirmed by using another siRNA targeting the CD44 gene (Fig. S1). Taken together, our results suggested that CD44 may promote the collective migration through inducing lamellipodia extension in leader cells.

### CD44–Ezrin interaction facilitates lamellipodia outgrowth in leader cells and drives the collective migration

**Figure 2:**
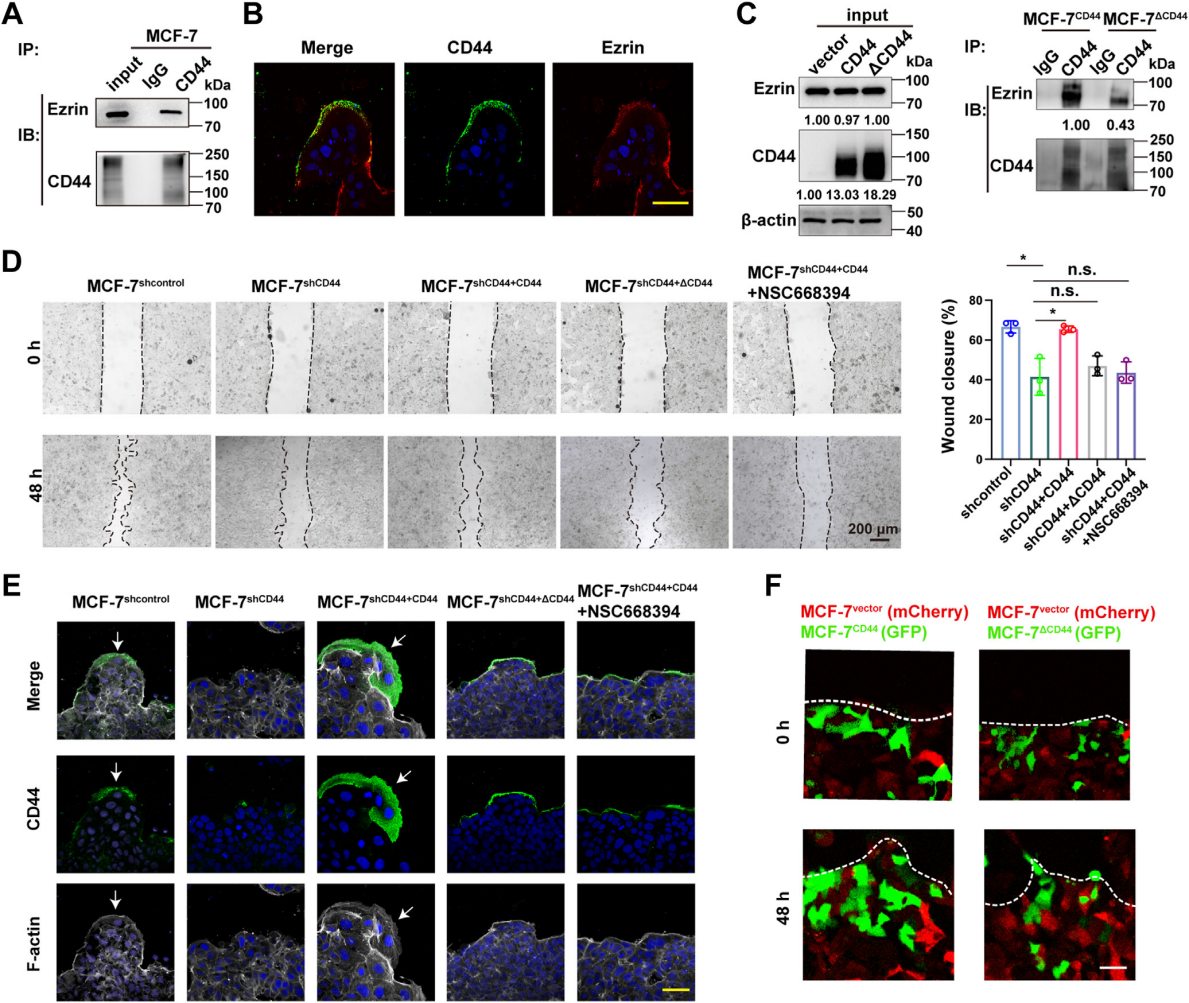
**CD44–Ezrin interaction facilitates lamellipodia formation in leader cells and drives the collective migration.***A*, Coimmunoprecipitation (Co-IP) analysis of CD44–Ezrin interaction in MCF-7 cells. *B*, immunofluorescence imaging analysis of colocalization of CD44 and Ezrin in the lamellipodia of MCF-7 cells. Scale bars represent 50 μm. *C*, co-IP analysis of Ezrin binding affinity between WT (WT CD44) and Ezrin binding–deficient CD44 mutant (ΔCD44). *D*, representative images of wound healing assay and quantitative analysis of wound closure rate (%) in control, shCD44, shCD44 + CD44, shCD44 + ΔCD44, and shCD44 + CD44 + NSC668394 MCF-7 cells. Wound closure rates (%) were presented as mean ± SD from three independent experiments. Scale bars represent 200 μm. Statistical significance: ns = not significant, ∗*p* < 0.05. *E*, immunofluorescence analysis of the distribution of CD44 (*green*) and F-actin (*white*) in control, shCD44, shCD44 + CD44, shCD44 + ΔCD44, shCD44 + CD44 + NSC668394 MCF-7 cells during collective migration. Scale bars represent 50 μm. *F*, representative fluorescence images of MCF-7 cells transduced with mCherry vector control, CD44-GFP, or ΔCD44-GFP in a competitive scratch wound assay (0 and 48 h postscratch). *White dashed lines* denoted wound boundaries. Scale bars represent 50 μm.

Next, we sought to elucidate the mechanism by which CD44 regulates lamellipodia formation and collective migration. A previous report demonstrated that CD44 induces cytoskeleton reorganization through the interaction of its cytoplasmic domain with Ezrin, a member of the ezrin–radixin–moesin protein family (22). We therefore wondered whether CD44-facilitated multicellular mobility was dependent on binding with Ezrin. Coimmunoprecipitation (IP) assays revealed a physical interaction between CD44 and Ezrin in MCF-7 cells, and confocal microscopic analysis further confirmed their colocalization at the lamellipodia in leader cells (Fig. 2, *A* and *B*). To functionally characterize the interaction, we transfected MCF-7 cells with either WT CD44 or an Ezrin binding–deficient CD44 mutant (ΔCD44). As expected, the ΔCD44 cells exhibited a reduced capacity for CD44–Ezrin binding (Fig. 2*C*).

In addition to the aforementioned results, we also performed rescue experiments in CD44-knockdown MCF-7 cells. Our results showed that overexpression of WT CD44 could restore the collective migration and lamellipodia formation in the leader cells, whereas the ΔCD44 mutant showed no restorative effects (Fig. 2, *D* and *E*). Moreover, treatment of WT CD44–rescued cells with the Ezrin inhibitor NSC668394 significantly impaired the cohesive migration and lamellipodia extension. Finally, competition migration assays indicated that WT CD44–expressing cells preferentially localized to the leading edge compared with ΔCD44-expressing cells (Fig. 2*F*). Collectively, these data suggested that the physical interaction between CD44 and Ezrin in the luminal-type BrCa leader cells could facilitate lamellipodia extension, leading to the collective migration.

### Sin3a is responsible for CD44 enrichment in the leader cells

**Figure 3:**
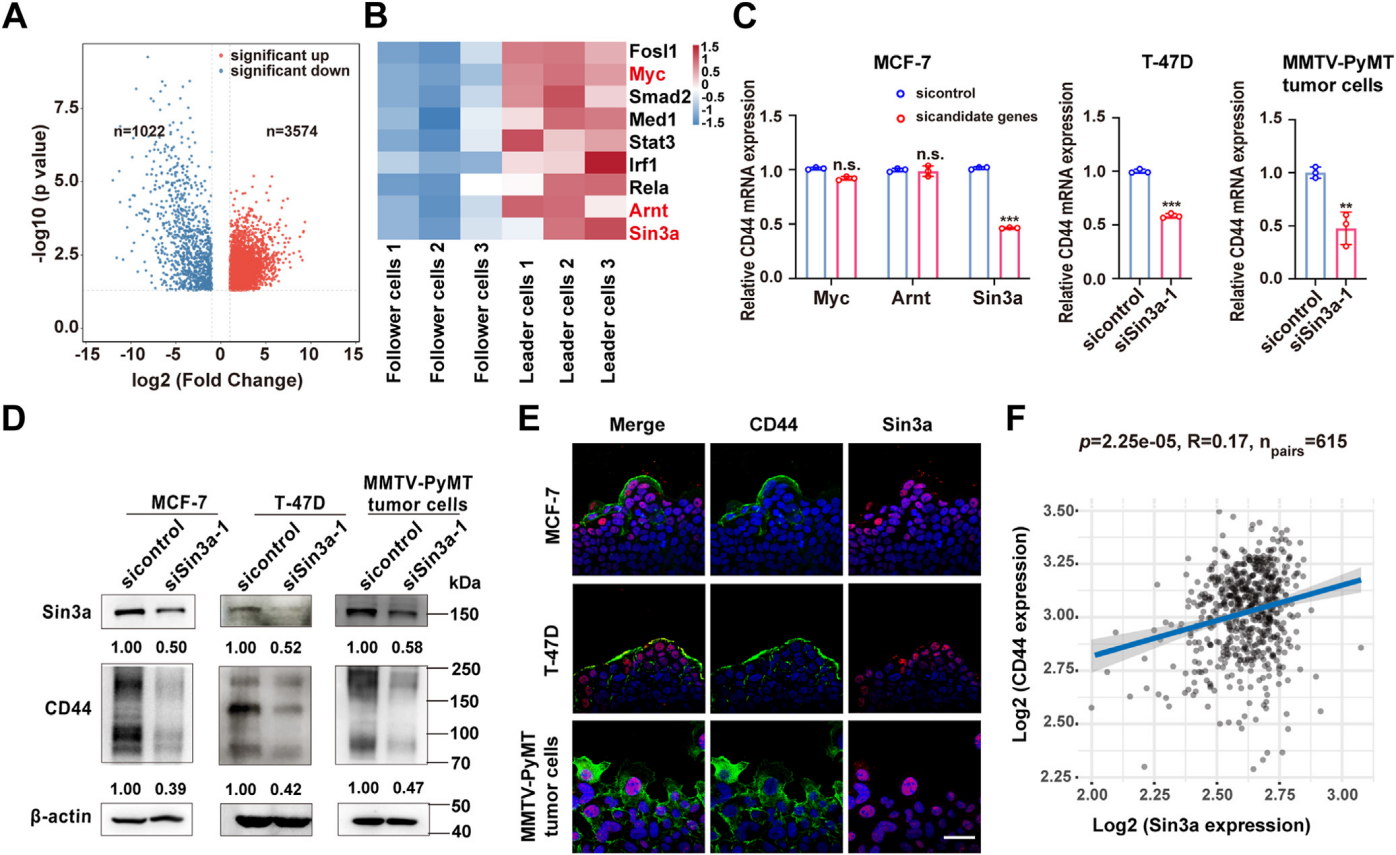
**Sin3a is responsible for CD44 enrichment in the leader cells of luminal-type BrCa cells.***A*, volcano plot showed differential gene expression (*p* value *versus* log2 fold change) in RNA-Seq analysis of CD44-high *versus* CD44-low expressing MMTV-PyMT tumor cells. *Vertical lines* represented 2.0-fold change cutoff, and the horizontal lines indicated the *p* value of 0.05. Significantly upregulated (*red*) and downregulated (*blue*) genes were highlighted. *B*, heatmap analysis of predicted CD44 transcriptional regulators in CD44-high *versus* CD44-low subpopulations in MMTV-PyMT tumor cells. Relative mRNA expression was represented by a color gradient (*red*: high expression; *blue*: low expression). *C*, quantitative RT–PCR analysis of CD44 expressions in control, Myc-knockdown, Arnt-knockdown, and Sin3a-knockdown MCF-7 cells, and in control *versus* Sin3a-knockdown T-47D and MMTV-PyMT tumor cells. Data were presented as mean ± SD (ns = not significant; ∗∗*p* < 0.01, ∗∗∗*p* < 0.001). *D*, Western blot analysis of CD44 and Sin3a expressions in control and Sin3a knockdown MCF-7, T-47D, and MMTV-PyMT tumor cells. *E*, immunofluorescence staining analysis of localization of CD44 (*green*) and Sin3a (*red*) in the collectively migrating MCF-7, T-47D, and MMTV-PyMT tumor cell clusters. Scale bars represent 50 μm. *F*, correlation scatter plot of CD44 and Sin3a expression levels in luminal BrCa specimens (n = 615) from The Cancer Genome Atlas (TCGA) database. Pearson correlation coefficient and significance were indicated. BrCa, breast cancer.

To identify the upstream regulators responsible for CD44 upregulation in leader cells, we purified CD44-high and CD44-low subpopulations from MMTV-PyMT tumors using fluorescence-activated cell sorting (FACS) and performed transcriptomic profiling. Differential expression analysis showed 3574 significantly upregulated and 1022 downregulated genes in leader cells compared with follower cells (fold change >2, *p* < 0.05, Figure 3*A*). Based on the results from hTFtarget (http://bioinfo.life.hust.edu.cn/hTFtarget#!/), we identified nine differentially expressed transcription factors as potential CD44 regulators (Fig. 3*B*). Further analysis of chromatin immunoprecipitation sequencing (ChIP-Seq) data from MCF-7 cells available in the public database Cistrome DB (http://cistrome.org/db/#/) suggested that Myc, Arnt, and Sin3a may bind to the CD44 promoter (Fig. 3*B*). To validate these findings, we examined the regulatory effects of the three candidates on CD44 expression. As illustrated in Figures 3, *C* and *D*, and S2, only the knockdown of Sin3a could downregulate both mRNA and protein levels of CD44. Of note, we observed colocalization of Sin3a protein with CD44 in the leader cells, further supporting its role as a key regulator in the activation of CD44 expression (Fig. 3*E*). In addition, analysis of 615 luminal BrCa tissue samples from The Cancer Genome Atlas database showed a positive correlation between Sin3a and CD44 expressions (Fig. 3*F*). Together, these data suggested that Sin3a may be responsible for CD44 enrichment in the leader cells.

### Sin3a activates CD44 transcription by binding to its promoter

**Figure 4:**
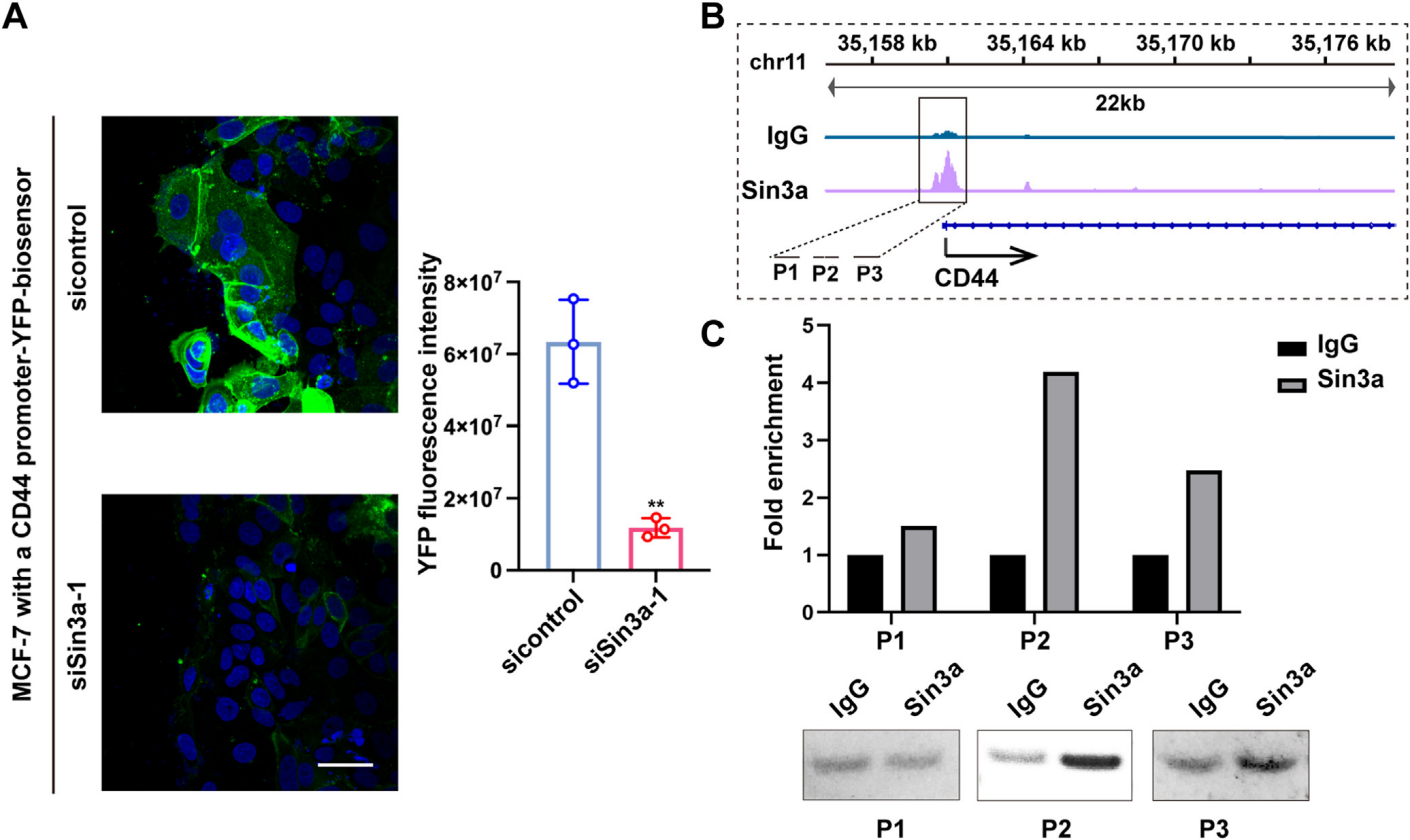
**Sin3a activates CD44 transcription by binding to the CD44 promoter.***A*, CD44 promoter activity assessed by YFP reporter expression in control *versus* Sin3a-knockdown MCF-7 cells. Quantification showed mean YFP fluorescence intensity ± SD from three independent experiments. Scale bars represent 50 μm. *B*, visualization of Sin3a ChIP-Seq binding profiles at the promoter of CD44 in MCF-7 cells. The *black box* indicated the CD44 promoter region targeted by three primers. *C*, ChIP–qPCR analysis of Sin3a enrichment at the promoter of CD44. ∗∗*p* < 0.01. ChIP-Seq, chromatin immunoprecipitation sequencing; qPCR, quantitative PCR.

As Sin3a is a transcription regulator (23), it is reasonable to speculate that Sin3a may directly bind to the promoter region of CD44. To this end, we engineered MCF-7 cells harboring a CD44 promoter biosensor where YFP serves as a reporter for CD44 gene transcription (7). As shown in Figures 4*A* and S3, the silence of Sin3a dramatically downregulated CD44 promoter activity in the leader cells. To investigate whether Sin3a physically interacted with the upstream promoter region of CD44, we performed the ChIP experiments. Based on the Sin3a ChIP-Seq data of MCF-7 cells available in the public database (GSE91789), we designed three primers specifically targeting the enrichment region of Sin3a within the CD44 promoter (Fig. 4*B*). ChIP was performed using either immunoglobulin G (IgG) (negative control) or Sin3a-specific antibodies, followed by RT–PCR analysis. The results indicated a substantial enrichment of the CD44 promoter region at the P2 site in Sin3a-immunoprecipitated chromatin compared with the IgG control (Fig. 4*C*). These findings suggested that Sin3a may bind to and activate CD44 promoter in the leader cells.

### Sin3a-induced CD44 activation in leader cells drives the collective migration

**Figure 5:**
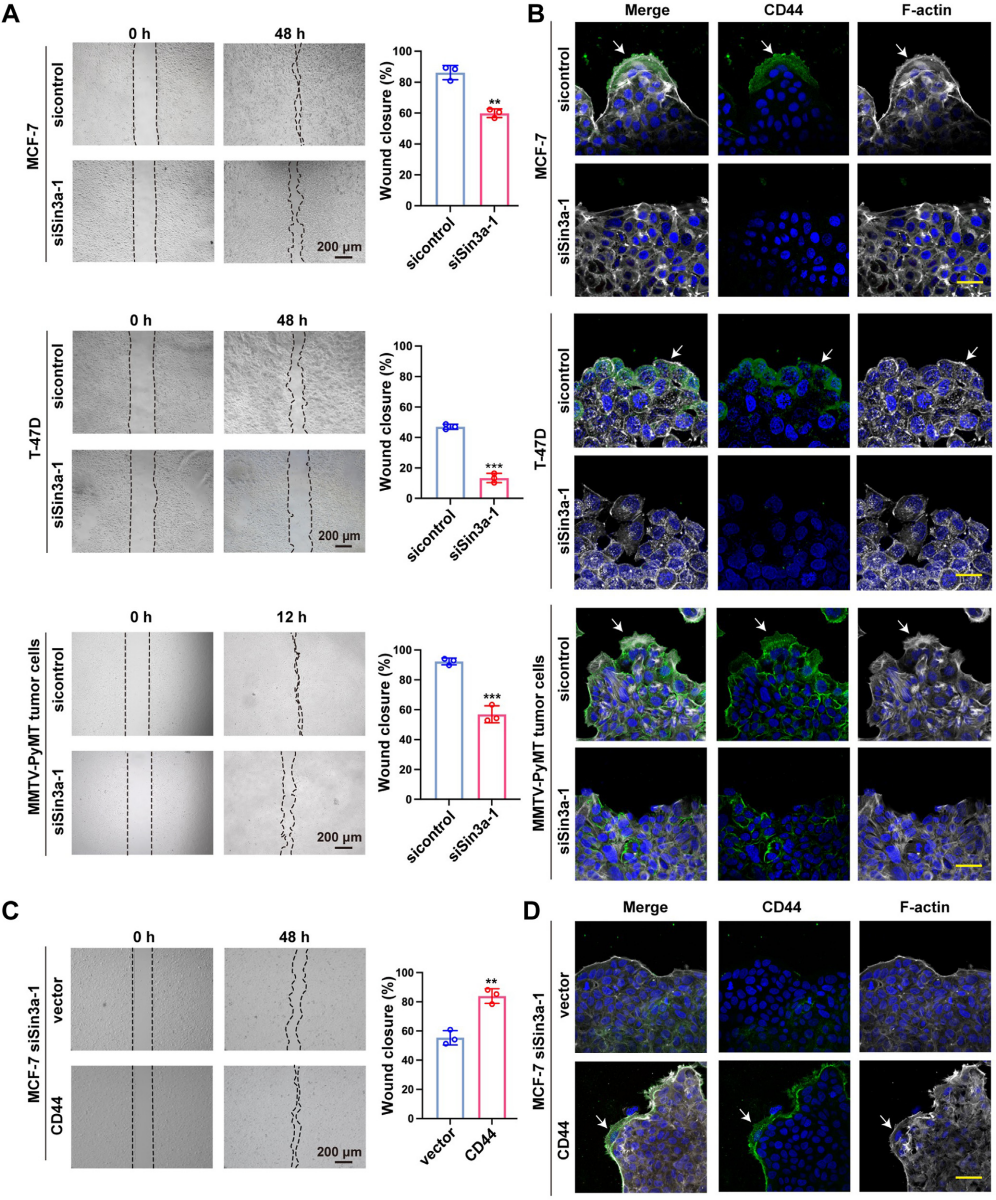
**Sin3a-induced CD44 activation in leader cells drives the collective migration of luminal-type BrCa cells.***A*, representative images and quantitative analysis of wound healing assays in control and Sin3a-knockdown MCF-7, T-47D cells, and MMTV-PyMT tumor cells. Wound closure rates (%) were presented as mean ± SD from three independent experiments. Scale bars represented 200 μm; ∗∗*p* < 0.01, ∗∗∗*p* < 0.001. *B*, immunofluorescence analysis of CD44 (*green*) and F-actin (*white*) distribution in control and Sin3a-knockdown MCF-7, T-47D, and MMTV-PyMT cells in collective migration. Scale bars represent 50 μm. *C*, representative images and quantitative analysis of wound closure rate (%) in MCF-7 cells transfected with siSin3a + vector *versus* siSin3a + CD44. The mean ± SD of wound closure rates from triplicate experiments was plotted. Scale bars represent 200 μm; ∗∗*p* < 0.01. *D*, immunofluorescence staining showed the subcellular distribution of CD44 (*green*) and F-actin (*white*) in collectively migrating Sin3a-knockdown MCF-7 cells with or without CD44 overexpression. Scale bars represent 50 μm. BrCa, breast cancer.

Having found Sin3a as a direct transcriptional activator of CD44 in leader cells, we next attempted to determine the involvement of Sin3a in collective migration of luminal-type BrCa cells. As shown in Figures 5, *A* and *B* and S4, the knockdown of Sin3a reduced CD44 expression in the leader cells and impaired cell cluster mobility as well as lamellipodia outgrowth. To substantiate the contribution of CD44 in Sin3a-mediated collective migration, we performed rescue experiments by overexpressing CD44 in Sin3a-silenced MCF-7 cells. Our results demonstrated that CD44 upregulation could restore the cooperative cell migration and lamellipodia outgrowth inhibited by Sin3a knockdown (Fig. 5, *C* and *D*). Together, our study indicated that Sin3a-induced CD44 activation could drive the collective migration of BrCa cells.

### Sin3a–CD44 axis facilitates luminal-type BrCa cell metastasis in zebrafish

**Figure 6:**
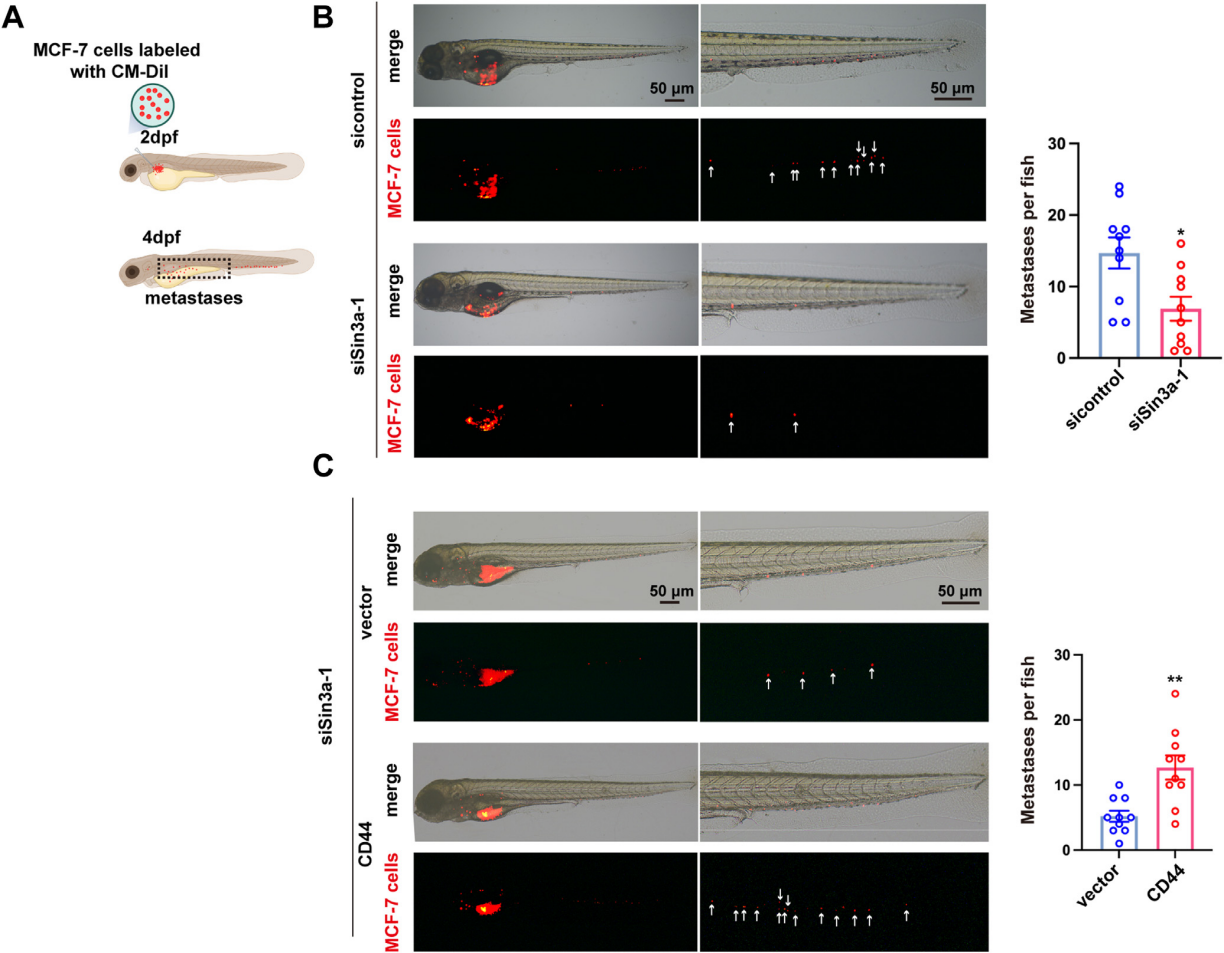
**Sin3a–CD44 axis facilitates luminal-type BrCa cells metastasis in zebrafish.***A*, schematic diagram of the zebrafish embryo xenograft assay (created with BioRender.com). CM-Dil-labeled control and Sin3a-knockdown MCF-7 cells were microinjected into the perivitelline space at 2 days postfertilization (dpf). Metastatic cells were observed by fluorescence microscopy at 4 dpf. *B*, quantification of metastatic potential in Sin3a-knockdown MCF-7 cells *versus* controls, n = 10. *C*, metastasis analysis of siSin3a + vector *versus* siSin3a + CD44 MCF-7 cells. Quantification of tumor foci shows mean ± SD (n = 10 embryos; ∗*p* < 0.05, ∗∗*p* < 0.01). BrCa, breast cancer.

To further validate the role of Sin3a–CD44 axis in luminal BrCa metastasis *in vivo*, we employed a zebrafish xenograft model. Briefly, CM-Dil-labeled MCF-7 cells (control or Sin3a knockdown) were microinjected into the perivitelline space of 2-day-old zebrafish larvae. Metastatic cells were observed by fluorescence imaging 2 days postinjection (Fig. 6*A*). As shown in Figure 6*B*, the knockdown of Sin3a could remarkably reduce the metastatic potential of MCF-7 cells in zebrafish larvae. Consistent with the *in vitro* results, CD44 overexpression restored the metastatic impairment induced by Sin3a knockdown *in vivo* (Fig. 6*C*). These data implied that the Sin3a–CD44 axis might play a significant role in promoting BrCa cell metastasis.

## Discussion

Cancer cells can migrate either individually or collectively as multicellular groups (3). During collective invasion, the leader cells at the front edge can drive the movement of the following ones generally observed in luminal type but not in triple-negative BrCa cells (5, 24). Although several studies have been carried out to investigate the mechanisms of collective migration, it remains unclear how the leading subpopulations contribute to the process (3). It has been reported that multicellular movement is dependent on the increased actin-rich protrusions, like lamellipodia, in leader cells (21). Besides, some transmembrane receptors have been identified at protrusions or pseudopodia that involve in pseudopodia formation (14, 25). However, their roles in the context of collective migration have not been fully elucidated. We have demonstrated that CD44 is enriched in the leader cells of luminal-type BrCa (7). In this study, we sought to investigate the involvement of CD44 in collective migration and the mechanisms underlined.

It is well accepted that CD44 is crucial in regulating cancer metastasis. For example, a previous study has shown that CD44 is concentrated in tumor cells at the invasive front of head and neck squamous cell carcinomas and promotes metastasis (26). However, the role and mechanisms of CD44 in collective migration are still obscure. In this article, we demonstrated that CD44 was predominantly localized in the lamellipodia of leader cells. Lamellipodia formation usually arises from cytoskeleton reorganization at the invasive front and is a prerequisite for cell migration (21). However, few studies have investigated the role of CD44 in lamellipodia outgrowth, particularly in the context of BrCa collective migration. To explore this, we focused on Ezrin, a cytoskeletal linker protein that involves in cytoskeleton reorganization through interacting with the cytoplasmic domain of CD44 (27). Our data indicated that the binding between CD44 and Ezrin could induce lamellipodia formation through the cytoskeletal rearrangement in leader cells. This result was further confirmed by blocking CD44–Ezrin interaction using a CD44 mutant with a disrupted Ezrin-binding site.

Given the significance of CD44 in collective migration, we next sought to investigate how CD44 becomes enriched in leader cells. In fact, it is well documented that transcription factors, such as p53, p63, Ets1 and Klf4, could regulate CD44 expression under various pathological conditions (19, 20, 28). However, the regulation of CD44 overexpression at the leading edge within the BrCa tumor mass remains largely unexplored. Here, we found that Sin3a was markedly upregulated in CD44-high expressing cells by transcriptomic sequencing analysis of MMTV-MyPT tumor cells. Importantly, we confirmed that Sin3a could bind to the CD44 promoter and subsequently upregulate the CD44 activation. As showed before, Sin3a is recognized as a transcriptional repressor that interacts with histone deacetylase HDAC1/2 and negatively regulates the expressions of proteins, such as p53, Ret, BMP7, and PRDM1 (29, 30, 31, 32). Also, Sin3a could act as a positive regulator by interacting with DNA demethylase Tet1 to promote demethylation and activate target genes (33). Whether our results that the transcriptional activation of CD44 in leader cells by Sin3a was dependent on DNA demethylases needs further investigation. In an effort to study the contribution of Sin3a-induced CD44 activation to BrCa metastasis, we conducted loss- and gain-of-function experiments. We found that the collective migration of BrCa cells was reduced by the knockdown of Sin3a, which could be rescued by the overexpression of CD44. Then, we injected the constructed cells into a zebrafish xenograft model and found that the metastatic potential was significantly decreased. These findings may highlight the significance of Sin3a–CD44 signaling axis in luminal-type BrCa metastasis.

**Figure 7:**
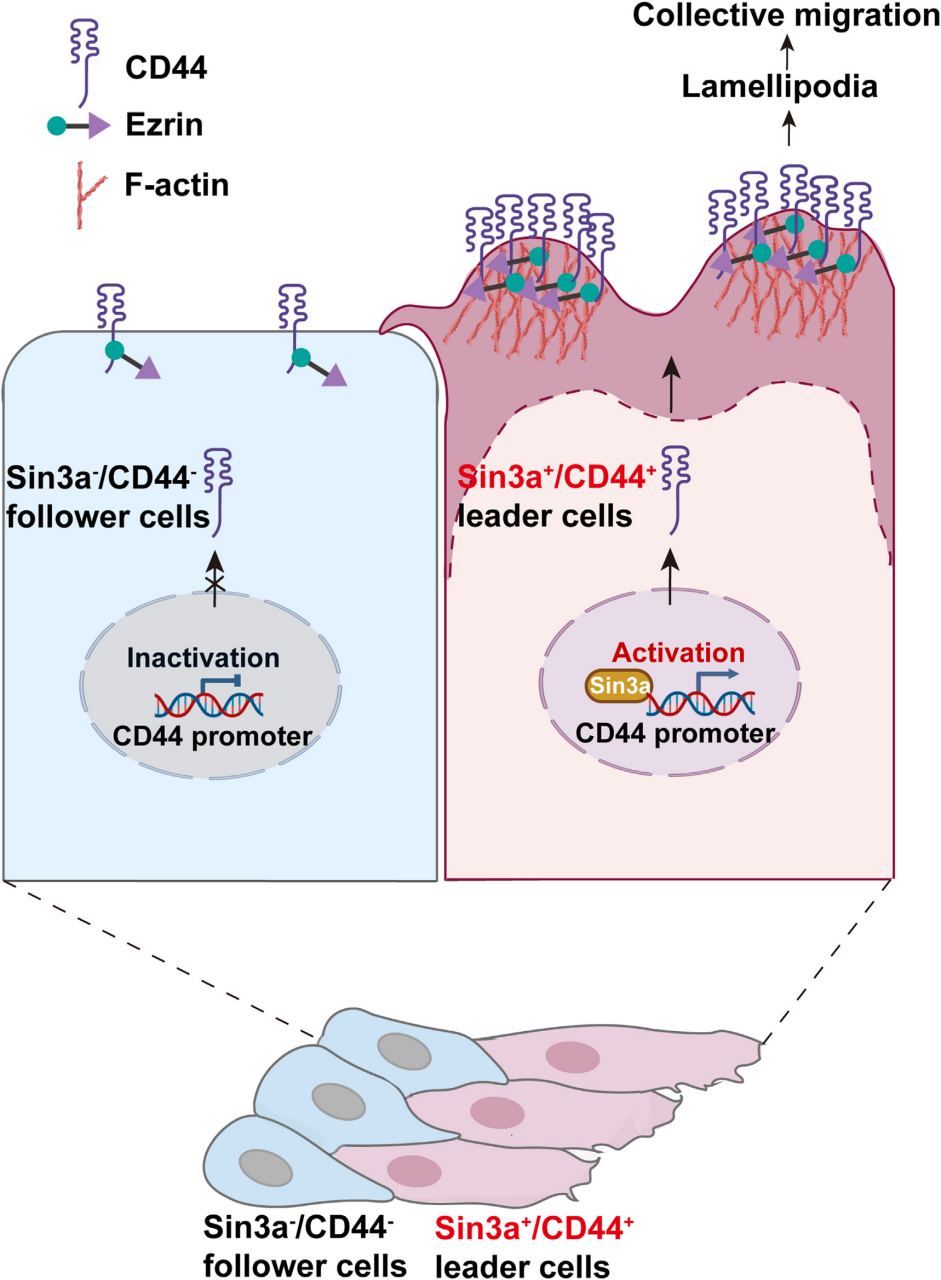
**Schematic illustration of the role of Sin3a in activating CD44 and promoting collective migration of luminal-type BrCa cells.** The diagram summarized the proposed mechanism by which Sin3a transcriptionally activated CD44 in leader cells. The interaction of CD44 with Ezrin could drive lamellipodia formation at the leading edge, leading to cohesive migration of luminal-type BrCa cells (created with BioRender.com). BrCa, breast cancer.

In summary, the present study identified that the upregulation of Sin3a in leader cells of BrCa could serve as a key regulator of CD44 transcriptional activation. Notably, the overexpressed CD44 could bind to Ezrin to induce lamellipodia formation, leading to collective migration (Fig. 7). Our findings revealed a mechanism for CD44 activation in driving the cohesive movement of luminal-type BrCa cells.

## Experimental procedures

### Cell culture and mice

The human luminal-type BrCa cell lines MCF-7 and T-47D were obtained from the American Type Culture Collection. All cell lines were authenticated through short tandem repeat DNA profiling and routinely tested for Mycoplasma contamination using a commercial detection kit (C0301S; Beyotime), with no contamination throughout the study. MCF-7 cells were cultured in minimum essential medium supplemented with 10% fetal bovine serum (FBS), 10 μg/ml insulin, 100 U/ml penicillin, and 100 μg/ml streptomycin. T-47D cells were cultured in RPMI1640 supplemented with 10% FBS, 7.5 μg/ml insulin, and the same antibiotic concentrations. All cells were incubated at 37 °C in a humidified atmosphere of 5% CO_2_ and routinely passaged at 80% confluency for experiments.

The MMTV-PyMT (FVB/NJGpt) transgenic mice were obtained from the Mode Animal Research Center of Nanjing University, China. All animal procedures were conducted in accordance with protocols approved by the Institutional Animal Care and Use Committee at Shanghai Jiao Tong University School of Medicine and performed under veterinary supervision. Primary tumor cells were isolated from the luminal-type mammary tumors of female MMTV-PyMT mice. Briefly, tumor tissues were minced and digested in PBS solution containing 1 mg/ml Liberase TL (05401020001; Roche) for 1 h at 37 °C with constant agitation. Following centrifugation, the cell suspension was treated with Hanks’ balanced salt solution containing 0.1 mg/ml DNase I (11284932001; Roche) and filtered through a 40 μm cell strainer (352235; BD). Then CD45^-^EpCAM^+^ tumor cells were purified by FACS (Beckman Coulter) and maintained in mammary epithelial cell basal medium supplemented with 10% FBS, 100 U/ml penicillin, and 100 μg/ml streptomycin.

### Antibodies and reagents

The following primary antibodies were used in this study: CD44 (ab189524; Abcam), β-actin (3700; Cell Signaling Technology [CST]), Sin3a (ab3479; Abcam; A01203-2, Boster), EpCAM-PE (12-9326-42; eBioscience), CD45-FITC (11-4714-42; eBioscience), CD44-FITC (555478; BD Pharmingen), and Ezrin (3145; CST). The Ezrin inhibitor NSC668394 (HY-115492) was obtained from MedChemExpress.

### siRNA, plasmids, and lentivirus

Control siRNAs and siRNAs targeting human/mouse CD44 and Sin3a were designed and synthesized by RiboBio. The transient transfections were carried out at 50 nM siRNA concentration using the riboFECT reagent according to the manufacturer’s instructions. Following a 15-min incubation of the transfection reaction mixtures at 20 to 25 °C, the mixtures were added to the cells. Culture media were replaced 6 h post-transfection, and the knockdown efficiency was validated at 72 h after transfection. The siRNA sequences were listed as follows: human siCD44-1 (5′-AUGCAAUGUGCUACUGAUUGU-3′), human siCD44-2 (5′-AGCTCTGAGCATCGGATTT-3′), mouse siCD44-1 (5′-GGAUCCGAAUUAGCUGGACACUCAA-3′), mouse siCD44-2 (5′-CACCUCCCAGUAUGACACAUU-3′), human siSin3a-1 (5′-GUGUGUCCCAGCUAUUCAATT-3′), human siSin3a-2 (5′-GGUGGAACAGAAUCGUUAUUU-3′), mouse siSin3a-1 (5′-CUACGUCUCAAGGAACCUTT-3′), mouse siSin3a-2 (5′-GGAUGGAUGAAGUAUAUAAUU-3′), and scramble negative control (5′-UUCUCCGAACGUGUCACGU-3′).

We utilized previously established CD44-knockdown MCF-7 cells to perform the following experiments (34). To generate the Ezrin binding–deficient CD44 mutant (ΔCD44), we introduced alanine substitutions at basic amino acid residues 292 to 300 (RRRCGQKKK→AAACGQAAA) in WT CD44 (NM_001001391) and cloned the mutant into the CS-Z0786-LX304-01 lentiviral vector (Genechem) (34). Lentiviral transduction was performed followed by selection with 2 μg/ml blasticidin (ST018; Beyotime) for 15 days to establish stable cell lines.

### Wound healing assay

Cell migration assays were performed using ibidi culture-inserts (80209; ibidi) in 35 mm μ-Dishes. Briefly, 4 × 10^4^ cells were seeded into each chamber of the two-well silicone insert and allowed to adhere for 24 h at 37 °C. Following insert removal, 2 ml fresh medium was added. Cell migration was monitored at 0, 12, and 48-h time points using phase-contrast microscopy, followed by fixation for immunofluorescence analysis. For competition scratch assays, differentially labeled MCF-7 cells were cocultured in a 1:1 ratio: vector control cells (red fluorescence) mixed with either WT CD44 or ΔCD44-expressing cells (green fluorescence). Following the removal of silicone insert, the spatial distribution of cells at the migration front was quantitatively analyzed using confocal microscopy (Nikon A1).

### Immunofluorescence

For immunofluorescence staining, cells were fixed in 4% paraformaldehyde for 20 min at 25 °C, followed by permeabilization with 0.2% Triton X-100/PBS for 10 min. Nonspecific binding sites were blocked with 5% bovine serum albumin for 1 h at 20 to 25 °C. Cells were then incubated with primary antibodies diluted in antibody dilution buffer (P0256; Beyotime) at 4 °C for 16 to 18 h. After three times of PBS washes, cells were incubated with secondary antibodies conjugated with Alexa Fluor 488 (ab150077; Abcam) or 594 (ab150080; Abcam) for 1 h at 20 to 25 °C. For cytoskeletal visualization, F-actin was stained with Phalloidin-iFluor 647 (ab176759; Abcam) for 1 h at 20 to 25 °C. Images were analyzed under confocal microscopy (Nikon A1).

### Coimmunoprecipitation

About 5 × 10^5^ cells (MCF-7^vector^, MCF-7^WTCD44^, and MCF-7^ΔCD44^) were lysed on ice for 30 min using radioimmunoprecipitation assay buffer (P0013J; Beyotime) supplemented with protease and phosphatase inhibitors. Cell lysates were incubated with 1 μg anti-CD44 antibody (ab119348; Abcam) or normal IgG control (2729; CST) at 4 °C for 16 to 18 h. The immune complexes were captured by incubation with 15 μl Protein A/G magnetic beads (88803; Thermo Fisher Scientific) at 4 °C for 2 h. Following three washes with cold lysis buffer, the immunoprecipitates were eluted and resolved by SDS-PAGE. Protein samples were then transferred to polyvinylidene fluoride membranes for subsequent Western blot analysis.

### Western blotting

Protein samples were extracted using radioimmunoprecipitation assay lysis buffer (P0013B; Beyotime) at 4 °C for 30 min. Protein concentrations were quantified using a bicinchoninic acid assay kit (23227; Sigma–Aldrich). Equal amounts of protein (20 μg) were separated by SDS-PAGE electrophoresis at 90 V and subsequently transferred to polyvinylidene fluoride membranes at 100 V for 1.5 h. Membranes were blocked with 5% nonfat milk in Tris-buffered saline plus Tween for 1 h at 20 to 25 °C with constant agitation, followed by 16 to 18 h incubation with primary antibodies at 4 °C. After washing, membranes were incubated with horseradish peroxidase–conjugated secondary antibodies for 1 h at 20 to 25 °C. Protein bands were visualized using enhanced chemiluminescence reagent (SQ201; Epizyme) and quantified using an ImageQuant LAS 4000 mini imaging system (GE). Protein expression levels were normalized to β-actin.

### RNA-Seq and data analysis

CD44-high and CD44-low subpopulations were isolated form MMTV-PyMT tumor cells by FACS. Total RNA was extracted and subjected to next-generation sequencing using the Illumina HiSeq X-10 platform (Illumina). Sequencing libraries were prepared following the manufacturer's protocol using the Illumina TruSeq RNA Sample Preparation Kit (Illumina). High-quality reads were aligned to the mouse reference mRNA (GRCh38) using Bowtie2. Gene expression levels were quantified as fragments per kilobase of transcript per million mapped reads using RNA-Seq by expectation maximization. Differential expression analysis was performed using the limma R package, with statistical significance defined as a fold change >2.0 and *p* < 0.05.

### Quantitative real-time PCR

Total RNA was extracted from the cultured cells using RNAiso Plus (9108; Takara Bio) according to the manufacturer's instructions. Then the RNA concentration was measured using NanoDrop system (Thermo Fisher Scientific, Inc). For complementary DNA synthesis, 1 μg of total RNA was reverse transcribed using the PrimeScript RT Reagent kit with gDNA Eraser (RR047Q; Takara Bio). The quantitative PCR assays were performed in triplicate using TB Green mix (RR820A; Takara Bio, Inc) according to the manufacturer's protocol. The thermal cycling protocol consisted of an initial denaturation at 95 °C for 5 min, followed by 40 cycles of 95 °C for 5 s and 60 °C for 34 s, with fluorescence acquisition at each cycle. Reaction specificity was confirmed by melting curve analysis. Cycle threshold values were collected with a cutoff of 35 cycles, and gene expression levels were normalized to the housekeeping gene *ACTB* using the 2-ΔΔCT method. Primer sequences are provided in Table S1.

### Chromatin immunoprecipitation

The ChIP assay was performed using a commercial Kit (P2078; Beyotime) following the manufacturer’s guidelines. Briefly, approximately 1 × 10^7^ MCF-7 cells per IP were crosslinked with 1% formaldehyde solution for 10 min at 20 to 25 °C, followed by quenching with 125 mM glycine. Cells were washed twice with ice-cold PBS and lysed in ChIP lysis buffer for 10 min on ice. Chromatin was sheared by sonication to generate DNA fragments ranging from 200 to 1000 bp. For IP, chromatin samples were diluted threefold in ChIP dilution buffer and incubated 16 to 18 h at 4 °C with 1 μg anti-Sin3a antibody (ab3479; Abcam) or normal IgG. Immune complexes were captured using Protein A/G agarose beads and sequentially washed with low-salt, high-salt, LiCl, and TE buffers. After elution in ChIP elution buffer, crosslinks were reversed by incubation at 65 °C for 4 h. DNA was purified using a DNA Fragment Purification Kit (9761; Takara) and analyzed by quantitative PCR with SYBR Green using gene-specific primers (Table S2). Enrichment was calculated relative to input DNA and normalized to IgG control.

### The zebrafish metastasis assay

The zebrafish WT AB strain was raised and maintained in a recirculating aquatic system at 28.5 °C under a 12:12 h light/dark cycle, in accordance with standard protocols, and fed with dry pellets twice a day. All experimental procedures were approved by the Institutional Animal Care and Use Committee. For the tumor metastasis model, 2-day postfertilization embryos were dechorionated and anesthetized with 0.15 mg/ml tricaine. Tumor cells were labeled with CM-Dil fluorescent dye (V22888; Invitrogen) for 30 min at 37 °C, washed, and resuspended in Hanks’ balanced salt solution containing 0.1 mg/ml DNase I. Approximately 400 labeled cells were microinjected into the perivitelline space of each embryo using a glass capillary needle. Injected embryos were maintained at 33 °C for 48 h, then anesthetized and imaged using fluorescence microscopy (Nikon; Eclipse Ts2R) at 4 day postfertilization to quantify metastatic cells.

### Statistical analysis

Statistical analysis was performed using GraphPad Prism 9.0.0 (GraphPad Software, Inc). Data were presented as mean ± SD. Comparisons between two groups were analyzed using unpaired Student's *t* tests, whereas multiple group comparisons were performed using one-way ANOVA with appropriate post hoc tests. Categorical data were analyzed using Fisher's exact test. A *p* value <0.05 was considered statistically significant.

## Data availability

The data that support the findings of this study are available in the *Experimental procedures* section and/or supporting information of this article.

## Supporting information

This article contains supporting information.

## Conflict of interest

The authors have no relevant financial or nonfinancial interests to disclose. The authors declare that they have no conflicts of interest with the contents of this article.
