# Supplementary material for: The transcriptional regulator Sin3a activates CD44 and promotes collective luminal breast cancer cell migration
Source: J Biol Chem. 2025 May 21;301(6):110264. doi: 10.1016/j.jbc.2025.110264 (PMC12206111; doi:10.1016/j.jbc.2025.110264)
Supplement: Supplementary material [file mmc1.docx]

**Supplementary table 1: The primer sequences for qPCR**

| Primers | Sequences (5’→3’) |
| --- | --- |
| Human CD44-qPCR forward | CTGCCGCTTTGCAGGTGTA |
| Human CD44-qPCR reverse | CATTGTGGGCAAGGTGCTATT |
| Mouse CD44-qPCR forward | CGGAACCACAGCCTCCTTTCAA |
| Mouse CD44-qPCR reverse | GTATCCTGATCTCCAGTAGGC |
| Human Sin3a-qPCR forward | ACCATGCAGTCAGCTACGG |
| Human Sin3a-qPCR reverse | CACCGCTGTTGGGTGATGA |
| Mouse Sin3a-qPCR forward | GAAGTCTCTGGATCACCAAGGC |
| Mouse Sin3a-qPCR reverse | CAGCGTTCTCTTCTGTAGCCTG |

**Supplementary table 2: The primer sequences for ChIP-PCR**

| Primers | Sequences (5’→3’) |
| --- | --- |
| Sin3a/CD44-promoter Chip forward 1 | CTCTGCGGGCTGCTTAGTC |
| Sin3a/CD44-promoter Chip reverse 1 | AAACAGTGACCTAAGACGGAGG |
| Sin3a/CD44-promoter Chip forward 2 | CAGGTTCGGTCCGCCATC |
| Sin3a/CD44-promoter Chip reverse 2 | AAACTTGTCCATGGTGTCCG |
| Sin3a/CD44-promoter Chip forward 3 | CCGGACACCATGGACAAGTT |
| Sin3a/CD44-promoter Chip reverse 3 | GGGCACTCACCGATCTGC |


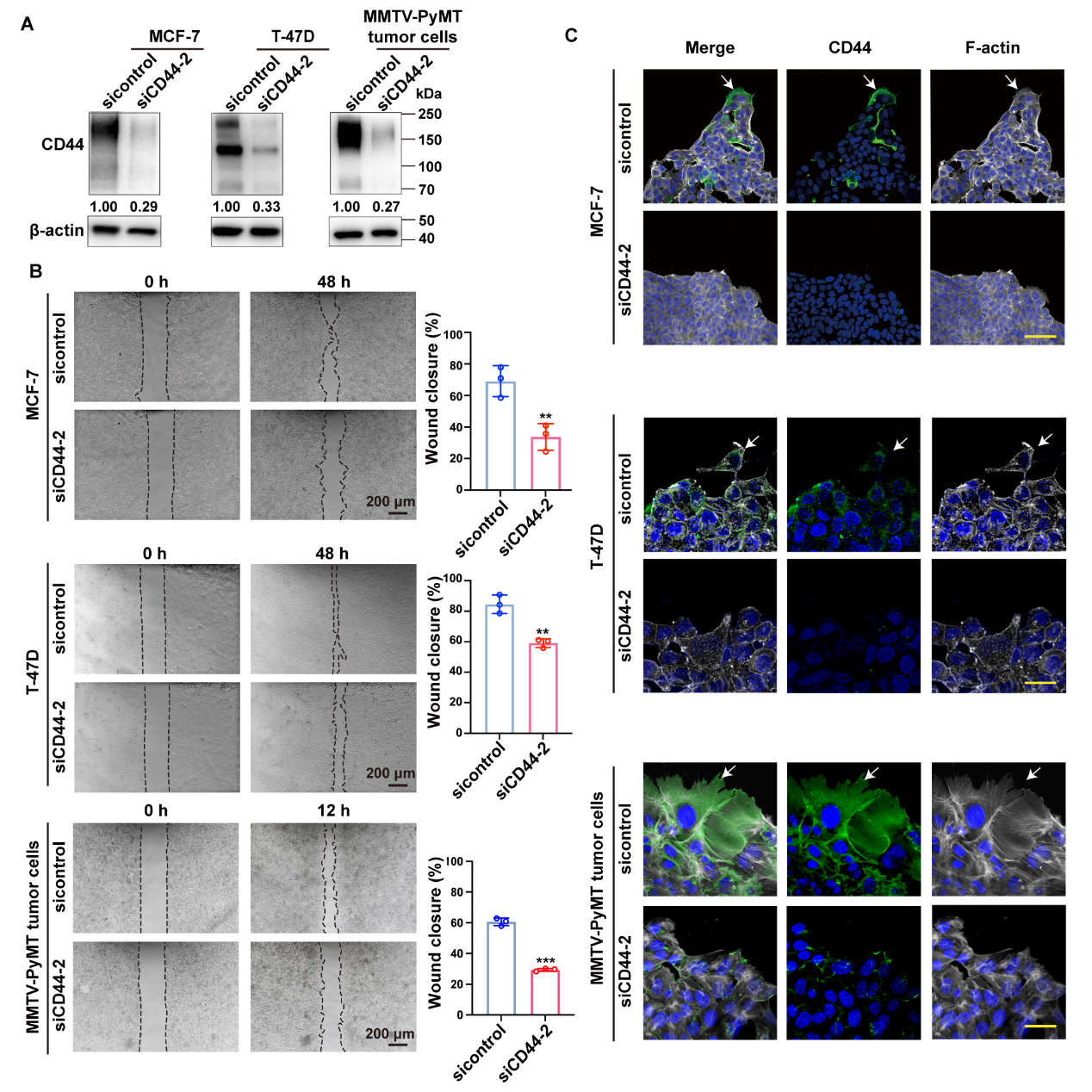


Fig.S1 CD44-enriched leader cells enhance collective migration in the luminal type BrCa cells

(A) Western blot analysis of CD44 levels in control versus CD44-knockdown MCF-7, T-47D cells and MMTV-PyMT tumor cells by using the second siRNA targeting CD44 (siCD44-2). (B) Representative images and quantitative analysis of wound healing assays in control versus CD44-knockdown MCF-7, T-47D cells and MMTV-PyMT tumor cells by using siCD44-2. Wound closure rates (%) were presented as mean ± SD from three independent experiments. Scale bars, 200 µm. ***p* < 0.01, ****p* < 0.001. (C) Immunofluorescence staining of CD44 (green) and F-actin (white) during collective migration in control versus CD44 knockdown MCF-7, T-47D cells and MMTV-PyMT tumor cells by using siCD44-2. Scale bars, 50 µm.


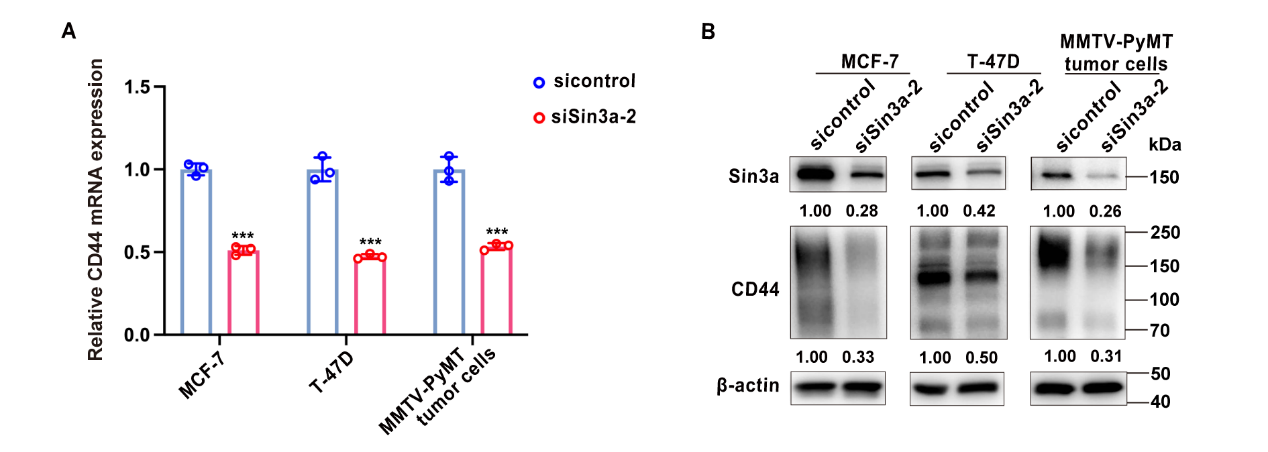


Fig.S2 Sin3a positively regulates CD44 expression in luminal type BrCa cells

(A) Quantitative RT-PCR analysis of CD44 expressions in control and Sin3a-knockdown MCF-7, T-47D cells and MMTV-PyMT tumor cells by using the second siRNA targeting Sin3a (siSin3a-2). Data were presented as mean ± SD (****p* < 0.001). (B) Western blot analysis of CD44 and Sin3a expressions in control and Sin3a knockdown MCF-7, T-47D and MMTV-PyMT tumor cells by using siSin3a-2.


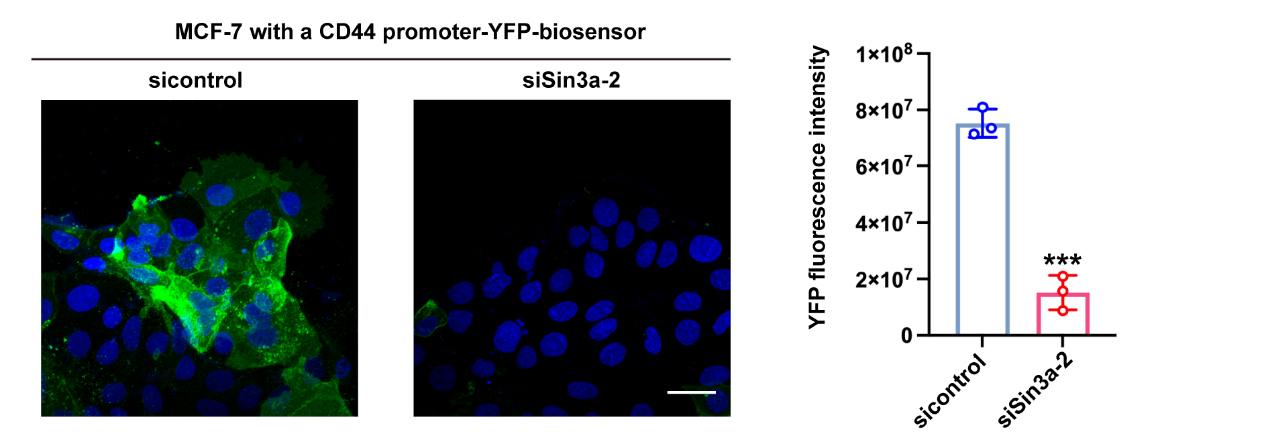


Fig.S3 Sin3a activates CD44 transcription in MCF-7 cells

(A) CD44 promoter activity assessed by YFP reporter expression in control versus Sin3a-knockdown MCF-7 cells by using siSin3a-2. Quantification showed mean YFP fluorescence intensity ± SD from three independent experiments. Scale bars, 50 μm. ****p* < 0.001.


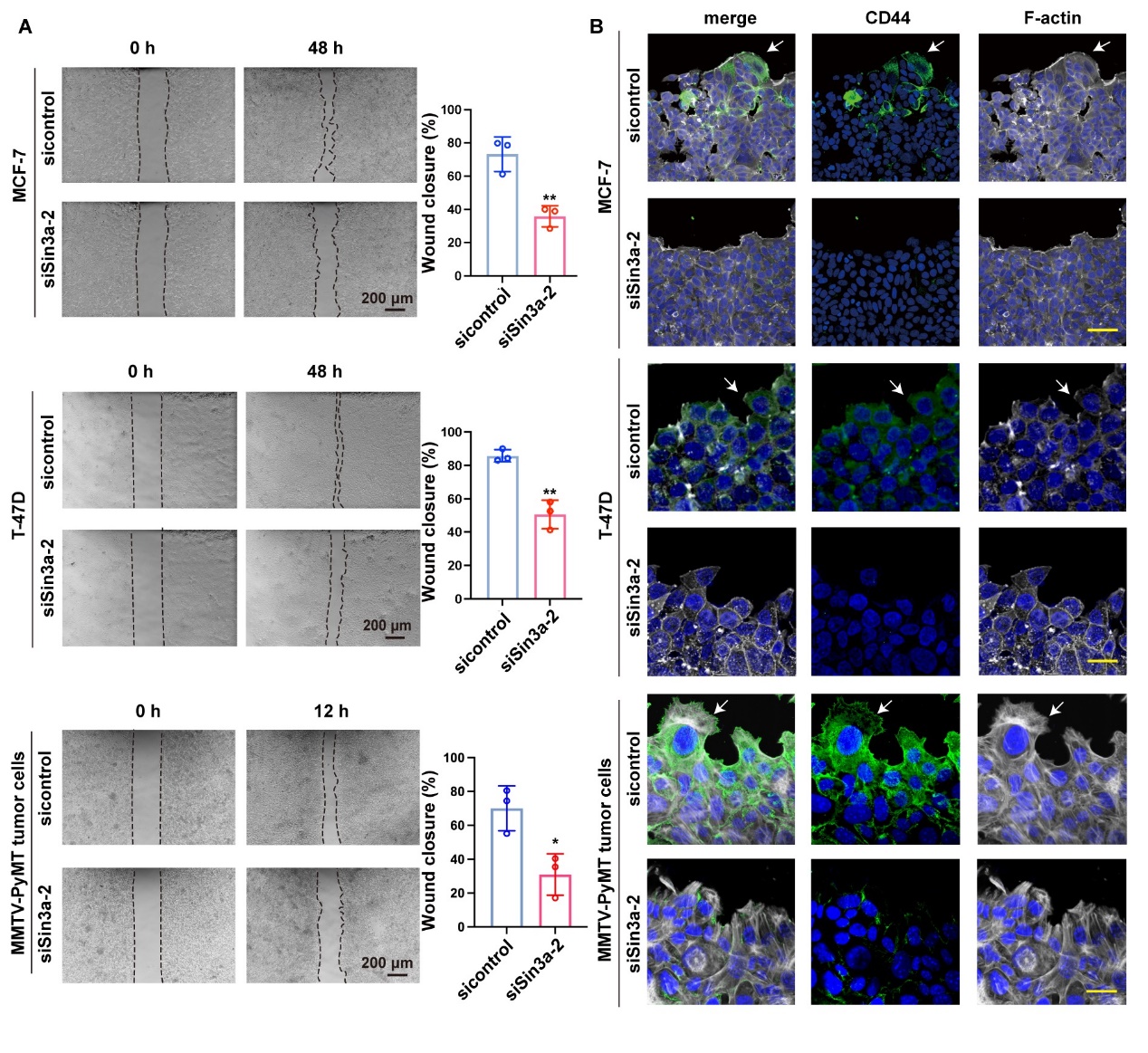


Fig.S4 Sin3a drives the collective migration of luminal type BrCa cells

(A) Representative images and quantitative analysis of wound healing assays in control and Sin3a-knockdown MCF-7, T-47D cells and MMTV-PyMT tumor cells by using siSin3a-2. Wound closure rates (%) were presented as mean ± SD from three independent experiments. Scale bars, 200 µm; **p* < 0.05, ***p* < 0.01. (B) Immunofluorescence analysis of CD44 (green) and F-actin (white) distribution in control and Sin3a-knockdown MCF-7, T-47D and MMTV-PyMT cells in collective migration by using siSin3a-2. Scale bars, 50 µm.
